# Supplementary material for: Differences in cancer patients’ work-cessation risk, based on gender and type of job: Examination of middle-aged and older adults in super-aged Japan
Source: PLoS One. 2020 Jan 29;15(1):e0227792. doi: 10.1371/journal.pone.0227792 (PMC6988938; doi:10.1371/journal.pone.0227792)
Supplement: S1 Appendix — (PDF) [file pone.0227792.s001.pdf]

Appendix

Average treatment effect on treated

Following Deheja and Wahba (1), we denoted the outcome (in this case, whether an individual will quit their job) of individual i as Y_{i1} if they were diagnosed with cancer, and Y_{i0} if they were not diagnosed with cancer, Hereafter, we refer to those who were diagnosed with cancer as the 'treatment' group. The treatment effect on a single entity, τ_i is defined as $\tau_i \equiv Y_{i1} - Y_{i0}$. We aimed to estimate the average treatment effect on the treatment group (ATT); that is,

$$\text{(S1.1)} \qquad ATT = E(Y_{i1}|T=1) - E(Y_{i0}|T=1)$$

$$= [E(Y_{i1}|T=1) - E(Y_{i0}|\ T=0)] + [E(Y_{i0}|T=0) - E(Y_{i0}|T=1)],$$

where T = 1, or 0 indicates the i th individual was assigned to the treated (control) group. If and only if the second term was zero, we could obtain a consistent and unbiased estimate of true ATT by simply comparing the means of the two groups. However, in our case, this assumption was unrealistic because cancer patients can potentially engage in unhealthy behaviours before diagnosis and, therefore, their counter-factual outcomes can differ from those of non-cancer patients.

To formalise the PSM estimator, we assumed several assumptions (observable covariates for person i are denoted as X_i).

[Assumption 1] (Unconfoundedness for controls)

$$\text{(S1.2)} \qquad \mathrm{Y_{i0}} \perp\!\!\!\perp T_i \ |\ X_i \Rightarrow E(Y_{i0}|X_i, T_i) = E(Y_{i0}|X_i)$$

[Assumption 2] (Overlap)

$$\text{(S1.3)} \qquad \Pr(T=1\ |\ X_i) < 1$$

These two assumptions are a weaker version of the 'strong ignorablity' defined by Rosenbaum and Rubin (2). Under these assumptions, ATT conditional on covariates, $ATT(X)$, was

$$\text{(S1.4)} \qquad ATT = E(Y_{i1}|X_i, T=1) - E(Y_{i0}|X_i, T=1)$$

$$= E(Y_{i1}|X_i, T=1) - E(Y_{i0}|X_i, T=0).$$

In the above equation, we could estimate ATT(X) by data. However, as highlighted in Caliendo and Kopeinig (3), ‘conditioning on all relevant covariates is limited in the case of a high dimensional vector X.’ To address this problem, we utilised the procedure in Rosenbaum and Rubin (2) and conditioned the probability of treatment as a function of covariates X, which is called ‘propensity score’. If the abovementioned assumptions were satisfied, Y_{i0} and T_i were independent conditional on the propensity score; that is, $Y_{i0} \perp\!\!\!\perp T_i \mid p(X_i)$ (Lemma 2.14 in reference (4)). Then, we constructed the ATT conditional on the propensity score, PSM, as follows:

$$\text{(S1.5)} \qquad \tau_{PSM} = E_{p(X)|\ T=1}\left[E(Y_{i1} \mid T = 1, p(X)\right) - E(Y_{i0} \mid T = 0, p(X))],$$

where $E_{p(X)|T\,=\,1}$ denotes the expectation of the treated population with regard to the propensity score, which implicitly claims the common support restriction.

Nearest Neighbour Matching

We employed nearest neighbour matching, in which a cancer patient was matched with a weighted average of the first 15 closest individuals in the control group (NN15). In this case, ATT could be obtained using the following formula (5):

$$\text{(S1.6)} \qquad \widehat{\tau_{PSM}} = \frac{1}{|T|} \sum_{i \in T} \left[Y_i^T - \sum_{j \in C(i)} w_{ij} Y_j^C \right],$$

where T denotes treatment group, $|T|$ is the number of entities in the treatment group, $C(i)$ indicates the subset of a control group matched with the treated i, and w_{ij} is weight on the j th individual. For detailed discussion regarding weighting strategies, see Heckman, Ichimura, and Todd (6).

In order to avoid bad matching, we set the following calliper restriction:

$$j \text{ is } \begin{cases} \text{included in } C(i) \text{ if } p_j \text{ is one of the 15 closest scores to } p_i \text{ and } ||p_i - p_j|| < c \\ \text{excluded otherwise} \end{cases}$$

As we note in the paper, we set $c = 0.01$. We computed the standard error for $\widehat{\tau_{PSM}}$ using bootstrapping with 200 replications.

Covariate Balancing Score

The covariate balancing scores before and after PSM in S1 Table.

S1 Table. Covariate balancing scores.

	Model 1								Model 2							
	Male				Female				Cognitive				Manual			
	Lagged		Simultaneous		Lagged		Simultaneous		Lagged		Simultaneous		Lagged		Simultaneous	
	U	M	U	M	U	M	U	M	U	M	U	M	U	M	U	M
LR χ^2	108.69	1.20	154.30	1.14	59.98	0.88	59.71	1.09	97.57	1.38	113.49	0.47	42.34	0.83	66.19	1.27
p-value	0.000	1.000	0.000	1.000	0.001	1.000	0.001	1.000	0.000	1.000	0.000	1.000	0.023	1.000	0.000	1.000
Mean% Bias	7.5	1.1	7.7	0.9	8.9	1.2	6.6	1.2	7.9	1.3	7.4	0.7	10.0	1.7	9.7	1.8
Med% Bias	5.5	0.8	4.5	0.8	7.1	0.5	4.6	1.2	6.1	1.2	5.4	0.5	8.4	1.7	6.9	1.7
Rubin's B	58.0*	8.4	55.5*	8.7	53.8*	9.5	43.3*	8.3	51.1*	8.5	44.6*	4.0	63.4*	12.4	59.7*	11.7
Rubin's R	0.84	0.99	0.95	0.88	1.37	0.93	1.10	1.03	0.93	0.95	1.03	0.93	0.92	0.91	0.82	0.93

Note: 'LR χ^2' indicates likelihood-ratio statistics of the joint insignificance of all covariates. 'P-value' indicates the corresponding p-values of the tests. Mean (Median)% bias is a summary indicator of the distribution of the standardised differences of the covariates after matching; generally, maximum bias at 10% is considered to signify negligible difference [11]. 'Rubin's B' is the absolute standardised difference of the means of the linear index of the propensity score in the treatment group and matched controls. 'Rubin's R' is the ratio of the treatment group to matched non-treated group in terms of the variances of the propensity score index. For sufficient balances, Rubin recommends that B be less than 25% and that R be between 0.5 and 2%. * if B > 25, R < [0.5; 2] [12,13].

Reference

1. Dehejia RH, Wahba S. Propensity score-matching methods for nonexperimental causal studies. *Rev Econ Stat* 2002; **84**: 151–161.
2. Rosenbaum PR, Rubin DB. The central role of the propensity score in observational studies for causal effects. *Biometrika* 1983; **70**: 41–55.
3. Caliendo M, Kopeinig S. Some practical guidance for the implementation of propensity score matching. *J Econ Surv* 2008; **22**: 31–72.
4. Imbens GW. Nonparametric estimation of average treatment effects under exogeneity: A review. *Rev Econ Stat* 2004; **86**: 4–29.
5. Becker SO, Ichino A. Estimation of average treatment effects based on propensity scores. *Stata J*

   2002; **2**: 358–377.
6. Heckman JJ, Ichimura H, Todd P. Matching as an econometric evaluation estimator. *Rev Econ Stud* 1998; **65**: 261–294.
7. Jenkins SP. Easy estimation methods for discrete-time duration models. *Oxf Bull Econ Stat* 1995;

   57: 129–136.
8. García-Gómez P, Jones AM, Rice N. Health effects on labour market exits and entries. *Labour Econ* 2010; **17**: 62–76.
9. Cox DR. Regression models and life-tables. *J Roy Stat Soc B Met* 1972; **34**: 187–222.
10. Meyer, B. Unemployment insurance and unemployment spells. *Econometrica* 1990; **58**: 757–782.
11. Austin PC. Balance diagnostics for comparing the distribution of baseline covariates between treatment groups in propensity-score matched samples. *Stat Med* 2009; **28**: 3083–3107.

12. Rubin DB. Using propensity scores to help design observational studies: application to the tobacco litigation. *Health Serv Outcomes Res Methodol* 2001; **2**: 169–188.

13. Leuven E. Help page of pstest command [document on the Internet, accessed November 24, 2018]. http://fmwww.bc.edu/repec/bocode/p/pstest.html.
