# Supplementary material for: Differences in cancer patients’ work-cessation risk, based on gender and type of job: Examination of middle-aged and older adults in super-aged Japan
Source: PLoS One. 2020 Jan 29;15(1):e0227792. doi: 10.1371/journal.pone.0227792 (PMC6988938; doi:10.1371/journal.pone.0227792)
Supplement: Attachment — Submitted filename: Response to Reviewers_Dec242019.docx [file pone.0227792.s002.docx]

**Response to the Reviewer #1**

We deeply thank the reviewer for the comments. Please kindly find below a detailed response to each of the comments**.**

- **Comment 1**

What is the difference between table 3 and figure 3? They contained almost the same information. The valuable data is estimation of ATT and 95% confidence of ATT. Author may consider the redundancy in data presentation.

**Response**

The reviewer is absolutely right. However, we dare to add Figure 3 for showing visually the readers how large the magnitudes of the effects are, as our research objective is to focus on the difference of job cessation risk between men versus women and/or cognitive versus manual workers.

- **Comment 2**

In table 3, the simultaneous ATT in female is 0.186. Does that really mean the female cancer patients were 18.6% more likely to quit their job? Or the relative risk is $e^{0.186}=1.204$. Then exact female cancer patients were 20.4% more likely to quit their job. I am not sure if I misunderstood the author’s reporting. Since ATT is the main concept in this study, author should add more detail process of data estimation as well as clinical implications. In addition, how big the ATT is denote clinical significant impact?

**Response**

We are sorry to make you confused. In our analyses, the estimated value indicates the effect measured by the percentage *point* (not percent) because the outcome is a dummy variable (1 if quitting the job; 0 otherwise). Let the probability of job cessation among the cancer patient and matched undiagnosed population be $P_{Diag}$ and $P_{NonDiag}$, then ATT can be interpreted as $P_{Diag}-P_{NonDiag}$. Because this is the difference between the two probabilities, this value should be interpreted as percentage points. If we want to derive the relative risk (RR) of job cessation between two groups, it can be computed by the simple equation: RR = $P_{Diag}/ P_{NonDiag}$. Moreover, because we use NN15 methodto implement the matching (which is one of the nearest neighbor matching where a cancer patient is matched with weighted average the first fifteen closest undiagnosed patients), we should note that $P_{NonDiag}$ is calculated as weighted average of matched samples. We add the sentences which clarify this regard in the manuscript as follows. (Please see the line 213-224)

“ATT is calculated as the difference in the probability of job cessation between people diagnosed as cancer and non-diagnosed population matched by PSM based on the covariates. If we denote them as $P_{Diag}$ and $P_{NonDiag}$, then

$$ATT= P_{Diag}- P_{NonDiag}.$$

Throughout the paper, nearest-neighbor matching was applied, through which each cancer patient was matched with a weighted average of the 15 closest non-cancer patients in terms of propensity score, and we imposed a caliper of 0.01 of the score to avoid poor matching balances. Therefore, $P_{NonDiag}$ is a weighted probability of job cessation of non-diagnosed and matched population, that is, $P_{NonDiag}= \sum_{i=1}^{15} w_{i}QUIT_{i}$, where $w_{i}$ is weight for each matched sample and $QUIT_{i}$ is a dichotomous variable indicating job cessations. Because ATT is the simple difference in two probabilities, ATT is measured by the changes in *percentage points*.”

Although RR can be computed by the abovementioned equation, the major problem of reporting RR is that we cannot report the standard error of the estimate. Given this, we should note that we add the result of the multivariable logistic regression and provide the results of the **odds ratio** between these two groups. Please see Table 5 in the manuscript.

In the logistic regression, the probability of job cessation ($p(quit)$) is assumed to follow the following function defined by K explanatory variables ($\mathbb{R}^{K}\to\mathbb{R}$), that is,

$$p\left( quit \right)= \frac{exp(\beta_{0}+\beta_{1}Diag_{it}+\boldsymbol{\gamma}\boldsymbol{X}_{\boldsymbol{it}}+\boldsymbol{\delta}\boldsymbol{\theta}_{\boldsymbol{t}}+\epsilon_{it})}{1+exp(\beta_{0}+\beta_{1}Diag_{it}+\boldsymbol{\gamma}\boldsymbol{X}_{\boldsymbol{it}}+\boldsymbol{\delta}\boldsymbol{\theta}_{\boldsymbol{t}}+\epsilon_{it})} ,$$

where $Diag_{it}$ is a dichotomous variable indicating the diagnosis of cancer, $\boldsymbol{X}_{\boldsymbol{it}}$ is a set of other controlled variables, $\boldsymbol{\theta}_{\boldsymbol{t}}$ is the year fixed effect, and $\epsilon_{it}$ denotes the random error term.

Considering that the odds are defined as $\frac{p\left( quit \right)}{1-p(quit)}$, the odds ratio between diagnosed and non-diagnosed population can be expressed as

$$OR= \frac{\frac{p\left( quit \right)}{1-p(quit)}| Diag=1}{\frac{p\left( quit \right)}{1-p(quit)}| Diag=0}=\frac{exp\left( log\left( \frac{p\left( quit \right)}{1-p(quit)}| Diag=1 \right) \right)}{exp\left( log\left( \frac{p\left( quit \right)}{1-p(quit)}| Diag=1 \right) \right)}= \frac{exp(\beta_{0}+\beta_{1}*1+\boldsymbol{\gamma}\boldsymbol{X}_{\boldsymbol{it}}+\boldsymbol{\delta}\boldsymbol{\theta}_{\boldsymbol{t}}+\epsilon_{it})}{exp(\beta_{0}+\beta_{1}*0+\boldsymbol{\gamma}\boldsymbol{X}_{\boldsymbol{it}}+\boldsymbol{\delta}\boldsymbol{\theta}_{\boldsymbol{t}}+\epsilon_{it})}=\exp\left( \beta_{1} \right).$$

Therefore, we suggest that $\exp\left( \beta_{1} \right)$ should be OR rather than ATT. We deeply thank you for providing an opportunity to discuss the interpretation of our estimate in detail.

Unfortunately, it is difficult to estimate the precise clinical impact because we do not have information about the types of cancer, treatment methods, or total cost spent on cancer treatment. In other words, our current study is partly able to capture the effects on *labor market* by estimating the probability of quitting the job; however, the resulting *clinical impact* remains unclear. We deeply apologize for not being able to respond to this comment in the current research. In the near future, we would like to reply to this important question after we get the opportunity to access the quality data in which the working status and clinical information can be identified.

- **Comment 3**

There is no mention about statistical method and software. Author should add more detailing process.

**Response**

We really appreciate you to point this out. We have declared the procedure of Propensity score matching in the supplementary material (please see the file named “Appendix.pdf”) in order to avoid a deviation of the “storyline” of the manuscript. All analyses were conducted by Stata MP 15.1. Please refer to the line 225-228 in the manuscript.

“Further rigorous discussion of PSM methodology, such as our matching quality, is shown in the web appendix [23,24]. All analyses (including Logistic regression) were performed using Stata MP 15.1. Almost all of the PSM results were performed by a user-written program, psmatch2.”

**Response to the Reviewer #2**

We deeply thank the reviewer for the comments. Please kindly find below a detailed response to each of the comments**.**

- **Comment 1**

In Table 2, there are some statistical difference in age, marital status, education level and so on. Although they adjusted these confounding factors in their analysis model, these factors might be important to decide to quit the job. Thus, further analysis will allow us the novel discussion in this study.

**Response**

We deeply thank you for your constructive suggestion. While these confounding factors are used as explanatory variables to implement PSM, it is possible to estimate the effect caused by these factors if we implement the multivariate regression model. To derive the odds ratio, we consider running logistic regression (the detailed procedure is declared in the subsection titled “Logistic regression” (p.11)). Table 6 in the revised manuscript reports the odds ratio of cancer diagnosis, gender, and types of jobs. In addition to this, we provide Table R2.1 (on the next page), as a supplementary analysis, in which the coefficients on age, marital status, health status, and education are reported.

**Table R2.1 Odds ratio of age, marital status, health status, and higher education**

|  | (1) | | (2) | |
| --- | --- | --- | --- | --- |
|  | Simultaneous | | One year lagged | |
|  | **OR** | **95% CI** | **OR** | **95% CI** |
| *Age* | 1.118*** | [1.107, 1.129] | 1.129*** | [1.117, 1.141] |
|  | (0.006) |  | (0.006) |  |
| *Marital status*  *(Reference: single)* |  |  |  |  |
| Married | 0.743*** | [0.657, 0.841] | 0.830*** | [0.722, 0.954] |
|  | (0.047) |  | (0.059) |  |
| Divorced/Widowed | 0.714*** | [0.622, 0.819] | 0.691*** | [0.592, 0.807] |
|  | (0.050) |  | (0.055) |  |
| *SRH*  *(Reference: excellent)* |  |  |  |  |
| good | 1.082 | [0.960, 1.219] | 0.972 | [0.859, 1.100] |
|  | (0.066) |  | (0.061) |  |
| comparatively good | 1.203*** | [1.069, 1.354] | 1.105 | [0.978, 1.248] |
|  | (0.073) |  | (0.069) |  |
| comparatively bad | 1.430*** | [1.249, 1.637] | 1.212*** | [1.050, 1.398] |
|  | (0.099) |  | (0.089) |  |
| bad | 1.722*** | [1.416, 2.092] | 1.524*** | [1.226, 1.895] |
|  | (0.171) |  | (0.169) |  |
| very bad | 2.641*** | [1.847, 3.775] | 1.418 | [0.871, 2.308] |
|  | (0.481) |  | (0.352) |  |
| *Education*  *(Reference: < univ.)* |  |  |  |  |
| ≥univ. level | 1.148*** | [1.071, 1.231] | 1.115*** | [1.034, 1.202] |
|  | (0.041) |  | (0.043) |  |
| Observation | 107,899 | | 88,955 | |
| Log Likelihood | -24535.08 | | -20252.01 | |

*Note*: Robust standard errors for heteroscedasticity are reported in parentheses.

Inference: *** p < 0.01; ** p < 0.05; * p < 0.1.

The results of Table R2.1 show some interesting implications. Firstly, aging would increase the probability of quitting job, which is highly consistent with our intuition. The second finding is that married or divorced people would be less likely to quit their job compared to single people. This may be because they have to support the dependents. With regard to health status, we can see the trend that poorer health would lead to higher risk of job cessation in the simultaneous setting. On the other hand, in the one year lagged model, the odds ratio of SRH being “very bad” is steeply decreased and becomes insignificant, which suggests that people who rate their health as extremely poor tend to quit their job immediately. This trend is highly parallel to the female and manual workers who are diagnosed as cancer. Finally, our result suggests that higher education leads to higher probability of job cessation. This result can be attributed to the fact that people who obtain university degrees tend to work as full-time worker and their retirement age is generally 60 to 70 years old. Therefore, when we use the sample aged 50-59, the significant effect disappears.

- **Comment 2**

Although they mentioned in study limitation, these results might be affected by severity of diseases, choice of treatment (surgery or medicine) or several medical aspects. In terms of clinical viewpoint, this analysis is insufficient to reach the conclusion.

**Response**

We totally agree with you. As we declared in “Limitation”, it is entirely impossible to identify the types of cancer and the severity of them. Therefore, we admit that our study cannot derive the *clinical* implication; however, please note that our primary objective in this paper is to investigate the effects on the *labor market*. We add the lines in which declare this point. Please see line 433-435.

“Of course, this limitation hinders us from deriving the implication from the clinical viewpoint; however, our study captures the average effect of cancer diagnosis on the labor market by focusing on the risk of job cessation.”

- **Comment 3**

It is interesting to find the gender gap and job-type gap in the analysis. Could you add the further analysis, such as male cognitive worker vs. male manual workers and female cognitive worker vs. female manual workers.

**Response**

We appreciate your comment. Please see Table R2.2 and R2.3 in which we investigate the effects among male cognitive, male manual, female cognitive, and female manual workers.

**Table R2.2 Simultaneous model**

|  | Cognitive | |  | Manual | |
| --- | --- | --- | --- | --- | --- |
|  | ATT | 95%CI |  | ATT | 95%CI |
|  |  |  |  |  |  |
| Male | 0.085*** | [0.047, 0.123] |  | 0.132*** | [0.061, 0.203] |
|  | (0.019) |  |  | (0.036) |  |
| Obs. | 35,655 |  |  | 14,515 |  |
|  |  |  |  |  |  |
| Female | 0.154*** | [0.092, 0.215] |  | 0.296*** | [0.153, 0.440] |
|  | (0.031) |  |  | (0.073) |  |
| Obs. | 28,976 |  |  | 5,938 |  |

*Note*: Bootstrapping standard errors with 200 replications are reported in parentheses.

Inference: *** p < 0.01; ** p < 0.05; * p < 0.1.

**Table R2.3 One year lagged model**

|  | Cognitive | |  | Manual | |
| --- | --- | --- | --- | --- | --- |
|  | ATT | 95%CI |  | ATT | 95%CI |
|  |  |  |  |  |  |
| Male | 0.047* | [-0.002, 0.097] |  | 0.045 | [-0.030, 0.120] |
|  | (0.025) |  |  | (0.038) |  |
| Obs. | 29,816 |  |  | 10,913 |  |
|  |  |  |  |  |  |
| Female | 0.005 | [-0.044, 0.054] |  | -0.053 | [-0.186, 0.080] |
|  | (0.025) |  |  | (0.068) |  |
| Obs. | 23,494 |  |  | 3,670 |  |

*Note*: Bootstrapping standard errors with 200 replications are reported in parentheses.

Inference: *** p < 0.01; ** p < 0.05; * p < 0.1.

From the above R2.2 and R.2.3, we can see that the trends suggested in the main manuscript hold even after we divide the sample. Male cognitive workers are likely to keep working after the diagnosis, while ATT for female manual workers amount to almost 30 percentage points. Obviously, these results are consistent to our conclusion in the article.

**Response to the Reviewer #3**

We deeply thank the reviewer for the comments. Please kindly find below a detailed response to each of the comments**.**

- **Comment 1**

Please add the cancer epidemiology in Japan.

**Response**

We add the following sentences. Please see the line 69-73.

“In Japan, as well as other developed countries, cancer has been and is one of the most critical issues in society and workplaces. Cancer accounts for almost 30% of total number of death in 2017 [8] and it has been the leading cause of death in Japan since 1981 [9]. Also, data in 2012 indicates that a third of total cancer patients were diagnosed as cancer when they were younger than 65 years old [10]. We should note that 325 thousand cancer patients regularly go to hospital for cancer treatment while they are working [10].”

More thorough and detailed cancer statistics in japan can be found in the link; <https://ganjoho.jp/data/reg_stat/statistics/brochure/2018/cancer_statistics_2018.pdf>

- **Comment 2**

Please strength the significance and importance of this issue in the introduction section.

**Response**

To emphasize the important point of our study, we summarize the novelty as follows. Please see the line 115-119.

“Specifically, the importance of our study can be summarized as follows: not merely we clarify the relationship between cancer diagnosis and job cessation, but also we focus on the heterogeneity of the effects based on gender and types of job, applying the econometric strategy to identify the causal effect.”

- **Comment 3**

Are there preliminary data or previous related studies regarding the work-cessation in Japan, if possible, please add in the introduction section.

**Response**

We find a survey related to our study and add the following sentences. Please see the line 111-115.

“In Japan, a survey focusing on the cancer diagnosis and occupation was conducted in 2004, where 34% of people who were newly diagnosed as cancer reported that they quit their job or they were dismissed [10]. However, now that this survey becomes outdated, it is obvious that re-evaluating the impact is critical and the rigorous statistical analyses which we provide will help a deeper understanding of this issue.”

- **Comment 4**

Please add the research purpose at the end of the introduction section.

**Response**

We explicitly articulate the research purpose of the study at the end of the introduction. Please see the line 120-121.

“The research purpose of the present study is to investigate “how large is the effect of a cancer diagnosis on job cessation?” and “is there any gap between gender or job types?”

- **Comment 5**

Please revise the discussion section following the revising introduction and the findings of the current study compare or discuss with previous studies, in which cited in the introduction section.

**Response**

We add the following sentences to compare our results with the past survey. Overall, the risk of job cessation seems to be moderated; however, it emphasizes the gender/job type differences. Please see the line 388-391.

“Compared to the survey conducted in 2004 where 34% of newly diagnosed cancer patients had to quit their job or were dismissed [10], our study suggests that the working environment for cancer patients has become improved. However, even if this is the case, our result underlines the fact that the gender/ job type gap has not been solved.”

- **Comment 6**

Conclusion was general, please revise following the findings of this current study.

**Response**

We repeat the overall results of the study in “Concluding remarks” as follows. Please see the line 439-444.

“In the analyses conducted by PSM, we observe that the male (female) cancer patients were 5.0 (-0.4 but statistically insignificant) percentage points more likely to quit their job in the next year of being diagnosed with cancer, and 10.1 (18.6) percentage points more likely to quit during the year of diagnosis. Regarding the job type gap, the one year lagged effect on cognitive (manual) workers are 3.8 (2.1 but statistically insignificant) percentage points and the simultaneous effects amount to 11.6 (18.7) percentage points.”

We thank for your suggestions on the manuscript. Now, the manuscript has been improved and the story has become clearer.

- **Comment 7**

Abstract: please revise the results following the research purpose and revise the conclusion following the revising conclusion in the texts.

**Response**

Please see the revised manuscript. We add concrete research purpose and the exact results of our statistical analyses.

**Response to the Reviewer #5**

We deeply thank the reviewer for the comments. Please kindly find below a detailed response to each of the comments**.**

- **Comment 1**

There are several major errors for the statistical testing and evaluation. First, in Table 2, the comparisons between diagnosed vs. not diagnosed on different demographic variables were conducted using t-test. It is relevant for continuous variables such as age and income, but not correct for categorical variables such as marital status, SRH, etc. The suitable statistical test should be chisq test to compare categorical variable between two subgroups.

**Response**

In our analysis, categorical variables such as marital status or SRH are treated as multiple dichotomous variables. For instance, if SRH = {very good, good, fair, poor}, we made four dichotomous variables: SRH_1 = {1 if SRH is *very good* and 0 otherwise}, SRH_2 = {1 if SRH is *good* and 0 otherwise}, SRH_3 = {1 if SRH is *fair* and 0 otherwise}, SRH_4 = {1 if SRH is *poor* and 0 otherwise}. Even in this case, we need to conduct $\chi^{2}$ test for unmatched samples to evaluate the difference between the two groups as you pointed out. On the other hand, because the weighted average of the variables is compared after they are matched, $\chi^{2}$statistics do not make sense; therefore, we keep to report t-statistics when we compare the sample average after matching is conducted. We are deeply sorry for our inappropriate statistical analysis. Now, we replace the t-statistics with chi-square statistics if applicable. Please see Table 2 in the revised draft.

- **Comment 2**

 Second, to evaluate the work cessation between male and female, manual and cognitive, logistic regression model can be considered while gender, and type of job can be treated as predictors. The advantage is that, it will provide odd ratio estimation to evaluate the magnitude of the differences. Besides that, it can also incorporate other variables to conduct multivariable analysis which was not conducted in the study.

**Response**

We deeply appreciate your constructive suggestion. It should be interesting to see the result of logistic regression in which the work cessation risk between male and female or manual and cognitive workers is compared. We add this analysis as Table 6. In this analyses, all the variables defined in Table 1 are included as potential predictors of job cessation. From Table 6, we can observe that female workers and manual workers face a higher risk of quitting their job regardless of the cancer diagnosis; the magnitude is about 1.2-1.3 times for female workers and about 1.07 times for manual workers.

- **Comment 3**

 There are additional analyses can be conducted to explore the potential interactive effect between gender and other factors, and type of job and other factors, on work cessation.

**Response**

Because it is impossible to derive the effect caused by the interaction between multiple predictors in the framework of ATT, we report the results of multivariate logistic regression incorporating several potential interaction terms. Specifically, we consider the interaction with age, subjective rated health (SRH), K6 score, and household income. The following figures (Fig R5.1 - R5.4) show the predicted probability of job cessation based on the abovementioned interaction terms and its 90% confidence interval. In this analysis, we use the dataset constructed in the simultaneous setting (please see Fig 1.); therefore, the estimated probability can be interpreted as *the risk of job cessation at period* $t$ *given that he or she worked in the period* $t-1$.

**Fig R5.1. Interaction between {Gender, Types of Job} * {Age}**

**Fig R5.2. Interaction between {Gender, Types of Job} * {SRH}**

*Note: SRH = 1: excellent, 2 : good, ..., 6: very poor*

**Fig R5.3. Interaction between {Gender, Types of Job} * {K6 score}**

**Fig R5.4. Interaction between {Gender, Types of Job} * {Log of Monthly Income (unit: JPY 10,000)}**

- **Comment 4**

It would be interesting to explore the working cessation rate after diagnosis across different year period.

**Response**

As we mentioned in the manuscript, we can define 11 time-windows (2005**–**2006, 2006–2007, 2007**–**2008, …, 2015**–**2016) for the simultaneous model (line 171) and 10 time-windows (2005–2007, 2006–2008**,** 2007–2009, …, 2014–2016) for the one year lagged model (line 155). Therefore, we separate these time-windows into two parts and run logistic regression to compare the effect of a cancer diagnosis on the job cessation. Table R5.1 summarizes the result.

**Table R5.1 Risk of job cessation after the cancer diagnosis; time-horizon comparison**

|  | Simultaneous | |  | One Year Lagged | |
| --- | --- | --- | --- | --- | --- |
|  | (I)^$1^ | (II)^$2^ |  | (III)^$3^ | (IV)^$4^ |
| Diagnosis | 3.472*** | 2.785*** |  | 1.395 | 1.647*** |
|  | (0.469) | (0.314) |  | (0.309) | (0.282) |
| Observation | 57,125 | 50,774 |  | 50,659 | 38,296 |
| Log Likelihood | -11089.74 | -13366.41 |  | -10152.30 | -10027.70 |

*Note*: Robust standard errors for heteroscedasticity are reported in parentheses.

$1: 5 time windows (2005-2006, 2006-2007, 2007-2008, 2008-2009, 2009-2010) are used in (I).

$2: 6 time windows (2010-2011, 2011-2012, 2012-2013, 2013-2014, 2014-2015, 2015-2016) are used in (II).

$3: 5 time windows (2005-2007, 2006-2008, 2007-2009, 2008-2010, 2009-2011) are used in (III).

$4: 5 time windows (2010-2012, 2011-2013, 2012-2014, 2013-2015, 2014-2016) are used in (IV).

Inference: *** p < 0.01; ** p < 0.05; * p < 0.1.

We can observe from the first two columns of Table R5.1 that the risk of quitting their current job during the year they were diagnosed with cancer decreased over the 12 years (from 3.472 times to 2.785 times). On the other hand, the risk of quitting their current job in the following year of the diagnosis has increased in the same period. Combining these results, we can infer that work environments that allow cancer patients to keep working have been being cultivated.

- **Comment 5**

It would be interesting to know the major types of cancer that were diagnosed and their impact on work cessation.

**Response**

In Japan, the major types of cancer among men are stomach, colorectal, and lung cancer if they are aged 50 to 70 (age range observed in our dataset). On the other hand, breast cancer is highly dominant and the risk of colorectal cancer steeply increases among women in this age range.^[1]^ For more detailed information, please see <https://ganjoho.jp/data/reg_stat/statistics/brochure/2018/cancer_statistics_2018.pdf> (cancer statistics in Japan- 2018). Especially, page 17-27 summarizes the statistics of cancer sites.

As you pointed out, it is important to compare the job cessation risk between different types of cancer. However, unfortunately, we cannot identify the types of cancer due to the data limitation as stated in the subsection “Limitation”. (Please see line 430-433)

“Finally, due to the data limitation, we were not able to identify types of cancer. Because a prognosis would be divergent depending on types [30], our result should be interpreted with caution in that it does not control for these types.”

We deeply apologize for not being able to respond to this comment with specific estimation results in the current research. In the near future, we would like to reply to these important questions by utilizing the clinical dataset along with the longitudinal study.

1. <http://www.gender.go.jp/about_danjo/whitepaper/h30/zentai/html/zuhyo/zuhyo01-00-41.html>
